# From Compliance-Based to Collaborative Financial Governance: A Bibliometric-Systematic Review of Multi-Actor Accountability in Public Financial Management

**DOI:** 10.12688/f1000research.187613.1

**Published:** 2026-10-12

**Authors:** Simon Saimima, Andy Fefta Wijaya, Endah Setyowati, Benny Hutahayan

**Affiliations:** 1 Faculty of Administrative Sciences, Universitas Brawijaya, Malang, East Java, 65145, Indonesia

**Keywords:** Public Financial Management; Collaborative Financial Governance; Fiscal Transparency; Multi-Actor Accountability; Bibliometric-Systematic Review

## Abstract

**Background:**

Public financial management has increasingly moved beyond a narrow compliance-oriented perspective toward broader governance arrangements involving transparency, oversight, participation, digitalization, and multiple accountability actors. This study maps the development of research on public financial management and formulates a conceptual agenda for collaborative financial governance.

**Methods:**

A bibliometric-systematic literature review was conducted using Scopus-indexed journal articles published in English between 2022 and 2026. After database filtering and eligibility screening, 265 articles were retained for bibliometric performance analysis and science mapping using Biblioshiny and VOSviewer. A second-stage screening identified 39 studies with direct thematic relevance for the systematic review and in-depth full-text thematic synthesis.

**Results:**

Publication output increased markedly after 2023 and peaked in 2025. Fiscal transparency, local government, accountability, budget transparency, audit, oversight, participation, public spending, and digital openness emerged as dominant and interconnected themes. The systematic synthesis indicates a shift from primarily compliance-based approaches toward broader multi-actor accountability and identifies six recurring governance domains: compliance, audit, transparency, participation, digitalization, and accountability.

**Conclusions:**

Collaborative financial governance is conceptualized as an integrated governance architecture in which these six domains operate as complementary mechanisms. The framework extends conventional public financial management by linking institutional control, fiscal openness, stakeholder involvement, digital support, and accountability to strengthen the legitimacy, responsiveness, and effectiveness of public resource management.

## 1. Introduction

Public financial management is a domain of public administration because it determines how public resources are planned, allocated, spent, monitored, reported, and justified. Gourfinkel (2022) emphasizes that public-sector accounting and reporting reforms are essential because governments require information systems to support accountability. Financial governance is not merely a process of budget execution, but a mechanism for demonstrating responsibility, legitimacy, and responsiveness. Its importance increases in crises, where transparency, resilience, and financial sustainability are closely connected ( Markuts & Roberto, 2025).

Nevertheless, public financial management reform has often been approached through a compliance-based logic. Formal rules, procedures, reports, and audits remain necessary, but compliance alone cannot ensure substantive accountability. Bisogno and Cuadrado-Ballesteros (2022) argue that budget transparency in the digital era also depends on e-government and e-participation. Similarly, Lino et al. (2022) show that compulsory digital transformation may generate procedural adoption without organizational change, while Gootjes and de Haan (2022) demonstrate that fiscal rules are more effective when supported by adequate budget transparency.

Transparency, audit, and oversight are therefore central to financial governance. Chen and Ganapati (2023) find that transparency can reduce corruption when fiscal and electronic openness are combined with accountability and publicity. Budget transparency enables public actors to access, evaluate, and question financial decisions, and is associated with effective fiscal rules and financial sustainability ( Gootjes & de Haan, 2022; Bisogno & Cuadrado-Ballesteros, 2022). Audit likewise serves the public interest by supporting expenditure accountability and democratic governance ( Ferry et al., 2023). However, its effectiveness depends on independence, authority, and follow-up, because political hegemony may weaken auditor independence and audit quality ( Sumiyana et al., 2023), while audit outcomes remain important for evaluating local government financial management ( Mabelane et al., 2023).

Public financial governance also increasingly involves participation and multi-actor accountability. Participatory budgeting can strengthen sustainable governance by giving citizens influence over financial decisions ( Sinervo et al., 2024). E-participation can enhance budget transparency, while civil society, taxpayer engagement, and fiscal openness broaden accountability beyond government-auditor relationships ( Bisogno & Cuadrado-Ballesteros, 2022; van den Boogaard et al., 2022). Digital tools enable this transition: public data platforms can improve fiscal transparency ( Han et al., 2025), audit digitalization can influence budget planning ( Lino et al., 2023), and blockchain-based monitoring may strengthen local budgetary control ( Kenetey & Popesko, 2025).

Previous reviews have examined public financial management from distinct perspectives. Detthamrong et al. (2024) map major themes such as transparency, accountability, and digitalization, while Bartocci et al. (2023) focus on participatory budgeting and citizen involvement. Roberto et al. (2025) further show that New Public Financial Management research remains concentrated on accounting, reporting, and performance, with limited interdisciplinary integration. However, these reviews have not yet brought compliance, audit, transparency, participation, digitalization, and accountability together within a unified governance perspective. This study addresses that gap by positioning collaborative financial governance as an integrative conceptual agenda that connects formal financial control with institutional oversight, stakeholder participation, digital openness, and multi-actor accountability.

Accordingly, this study examines the development and intellectual structure of research on public financial management, fiscal transparency, budget transparency, accountability, audit, oversight, and participation between 2022 and 2026. It also seeks to integrate the bibliometric and thematic findings into a conceptual agenda for collaborative financial governance. To achieve these objectives, the study addresses four research questions:

RQ1: How has the literature on public financial management, fiscal transparency, budget transparency, accountability, audit, oversight, and participation evolved from 2022 to 2026?

RQ2: Who are the most influential authors, sources, countries, and documents in this research field?

RQ3: What dominant and emerging themes appear in the keyword co-occurrence network and thematic map?

RQ4: How can the bibliometric and thematic findings be integrated to develop a conceptual agenda for collaborative financial governance?

## 2. Literature Review

### 2.1 Public Financial Management And Financial Governance

Public financial management encompasses the planning, budgeting, execution, reporting, auditing, monitoring, and accountability of public resources. Reliable accounting and reporting systems are essential for improving transparency, accountability, and institutional capacity ( Gourfinkel, 2022). Financial governance extends this function by emphasizing openness, compliance, efficiency, effectiveness, oversight, and public interest. Budget transparency is associated with governance quality and financial sustainability, making it central to long-term fiscal management ( Bisogno & Cuadrado-Ballesteros, 2022; Cuadrado-Ballesteros & Bisogno, 2022). Transparency also strengthens the effectiveness of fiscal rules and improves budget efficiency by widening access to financial information ( Gootjes & de Haan, 2022; Jung, 2022). Audit and oversight further support public spending accountability and democratic governance by evaluating compliance, performance, independence, and institutional responsibility ( Ferry et al., 2023). Audit digitalization can influence local budget planning, while audit outcomes help assess local government financial statements ( Lino et al., 2023; Mabelane et al., 2023). Digital platforms may improve fiscal transparency and public access, but their contribution depends on whether they strengthen openness, oversight, and accountable decision-making rather than merely accelerate compliance-oriented administration ( Han et al., 2025; Lino et al., 2022; Zhu & Yu, 2024).

### 2.2 Collaborative governance and multi-actor accountability

Collaborative governance refers to a governance approach in which public issues are addressed through interaction, coordination, and participation among multiple governmental and non-governmental actors. Within public financial management, this perspective emphasizes that financial decision-making and accountability may involve not only government institutions but also audit bodies, legislatures, citizens, civil society, and other stakeholders. Participatory budgeting represents one form of such involvement by providing citizens with opportunities to participate in public financial decision-making ( Sinervo et al., 2024). Multi-actor accountability refers to accountability relationships that operate through vertical, horizontal, and social mechanisms. Vertical accountability concerns responsibility within formal governmental structures, horizontal accountability involves oversight among public institutions, and social accountability involves scrutiny by citizens and civil society ( Ferry et al., 2023; van den Boogaard et al., 2022). Participation may also take place through e-government, e-participation, and participatory budgeting mechanisms that facilitate public access and involvement in budgetary processes ( Bisogno & Cuadrado-Ballesteros, 2022; Shybalkina & Nabatchi, 2024). Transparency and oversight constitute related governance mechanisms that enable financial information and public decisions to be examined by institutional and societal actors ( Chen & Ganapati, 2023; Wang & Sun, 2026). In public financial governance, these accountability mechanisms may also be supported by audit institutions and digital monitoring systems ( Mukhamedyarova et al., 2025; Kenetey & Popesko, 2025).

## 3. Research Method

### 3.1 Research design

This study employed a bibliometric-systematic literature review design to examine the development of public financial management and related transparency-accountability literature from 2022 to 2026. Bibliometric analysis was used to map the quantitative structure of the field, including publication growth, influential sources, productive authors, leading countries, highly cited documents, keyword co-occurrence, and thematic development. Donthu et al. (2021) explain that bibliometric analysis is useful for examining large volumes of scientific data, identifying the evolution of a research field, and detecting emerging research areas. Therefore, this method is appropriate for identifying the intellectual and conceptual structure of literature on public financial management, fiscal transparency, budget transparency, accountability, audit, oversight, and participation.

The systematic literature review component was used to interpret the bibliometric findings and develop a conceptual synthesis. Zupic and Čater (2015) argue that bibliometric methods can improve objectivity in literature reviews, but the interpretation of bibliometric outputs still requires theoretical and substantive reading. In this study, the systematic review did not merely summarize individual articles, but synthesized the main themes emerging from bibliometric mapping into a conceptual agenda for collaborative financial governance. Mukherjee et al. (2022) emphasize that bibliometric research should move beyond descriptive mapping and contribute to theory development and practical understanding. Accordingly, the combination of bibliometric analysis and systematic review enabled this study to connect quantitative mapping with conceptual interpretation.

### 3.2 Data source

The data were obtained from Scopus because it provides structured metadata suitable for bibliometric analysis, including titles, abstracts, author keywords, index keywords, source titles, affiliations, countries, citations, and references. Aria and Cuccurullo (2017) note that comprehensive science mapping requires reliable bibliographic metadata, and Scopus is widely used in bibliometric studies because of its broad journal coverage and standardized indexing system. The use of Scopus was also aligned with the objective of this study, which focuses on peer-reviewed journal articles indexed in an international scholarly database. The dataset used in this study consisted of 265 final journal articles published in English between 2022 and 2026. All records were exported from Scopus in CSV format for bibliometric processing and thematic screening.

### 3.3 Search strategy

The literature search was conducted in the Scopus database from 4 to 7 July 2026, using the title, abstract, and keyword fields. The search string was designed to capture three main components: public financial management and public finance, transparency-accountability mechanisms, and public sector or government context. The following query was used:

TITLE-ABS-KEY (“public financial management” OR “public finance” OR “fiscal transparency” OR “budget transparency”) AND TITLE-ABS-KEY (accountability OR transparency OR audit OR oversight OR participation) AND TITLE-ABS-KEY (“local government” OR “public sector” OR government).

The search was then limited to journal articles, English language, final publication stage, and the publication period from 2022 to 2026. These restrictions were applied to ensure that the dataset reflected recent, peer-reviewed, and complete scholarly contributions. Page et al. (2021) emphasize that systematic review reporting should clearly describe the identification and selection process so that the review can be transparent and reproducible. For that reason, the search strategy, source database, publication period, document type, language, and publication stage were explicitly defined before data analysis.

### 3.4 Inclusion criteria

The inclusion criteria were established to ensure consistency between the research questions and the bibliometric dataset. Articles were included when they met six criteria. First, the article had to be indexed in Scopus. Second, the article had to be published between 2022 and 2026. Third, the document type had to be an article. Fourth, the language had to be English. Fifth, the source type had to be a journal and the publication stage had to be final. Sixth, the article had to be substantively related to public financial management, public finance, fiscal transparency, budget transparency, accountability, transparency, audit, oversight, participation, local government, public sector, or government.

### 3.5 Exclusion criteria

Several exclusion criteria were applied to maintain the relevance and quality of the dataset. Conference papers, book chapters, editorials, notes, errata, and other non-article documents were excluded because they did not match the document-type restriction. Non-English articles were excluded to maintain language consistency in metadata interpretation and thematic screening. Articles focusing purely on corporate finance, private accounting, or corporate governance were excluded because they were outside the scope of public financial management and public sector governance. Articles with incomplete metadata were also excluded because missing bibliometric information could affect citation analysis, keyword analysis, and source mapping. These exclusion criteria helped ensure that the final dataset was suitable for both bibliometric analysis and systematic thematic synthesis.

### 3.6 Data screening

The screening process followed the logic of the PRISMA framework. Page et al. (2021) explain that systematic reviews should report the flow of studies from identification to screening, eligibility assessment, and inclusion. In the identification stage, metadata were retrieved from Scopus using the predefined query and database filters. The screening process was conducted by four reviewers. At the screening stage, the reviewers examined the titles, abstracts, and keywords to determine whether the articles were related to public financial management, fiscal or budget transparency, accountability, audit, oversight, participation, and government or public sector contexts. The same four reviewers were also involved in the full-text eligibility assessment to confirm the substantive relevance of the articles to the study focus. Articles that did not meet the predefined criteria were excluded. In the inclusion stage, the final dataset was used for bibliometric analysis and systematic thematic synthesis.

### 3.7 Data cleaning

Data cleaning was conducted before bibliometric analysis to reduce keyword fragmentation and improve the accuracy of science mapping. Aria and Cuccurullo (2017) emphasize that bibliometric analysis requires systematic data preparation because inconsistent terms may distort co-word analysis and thematic mapping. In this study, several keyword variations were standardized. For example, “PFM” was standardized as “public financial management,” “public finances” as “public finance,” “fiscal transparency” and “budget transparency” as “fiscal/budget transparency” when needed for thematic interpretation, and “auditing,” “public audit,” and “audit” as “audit.” Similar standardization was applied to “citizen participation,” “public participation,” and “participation,” as well as “local governments” and “local government.” This cleaning process was intended to ensure that similar concepts were not separated into different nodes during keyword co-occurrence and thematic analysis.

### 3.8 Data analysis

Data analysis combined bibliometric analysis and systematic thematic synthesis. Bibliometric analysis comprised performance analysis and science mapping, which assess research productivity, influence, and relationships among research components ( Donthu et al., 2021). Biblioshiny generated descriptive outputs and thematic maps, while VOSviewer visualized keyword co-occurrence networks ( Aria & Cuccurullo, 2017). Afterward, thematic synthesis interpreted the clusters, as co-word analysis alone cannot fully explain conceptual meaning ( Zupic & Čater, 2015). The synthesis focused on compliance-based financial governance, accountability and transparency, participation and oversight, and local government financial governance, which were integrated into a collaborative financial governance agenda aligned with the four research questions.

To ensure a transparent and reproducible literature selection process, the identification, screening, eligibility assessment, and final inclusion stages were structured according to the PRISMA framework. The complete study-selection pathway is presented in Figure 1.

**Figure 1.**
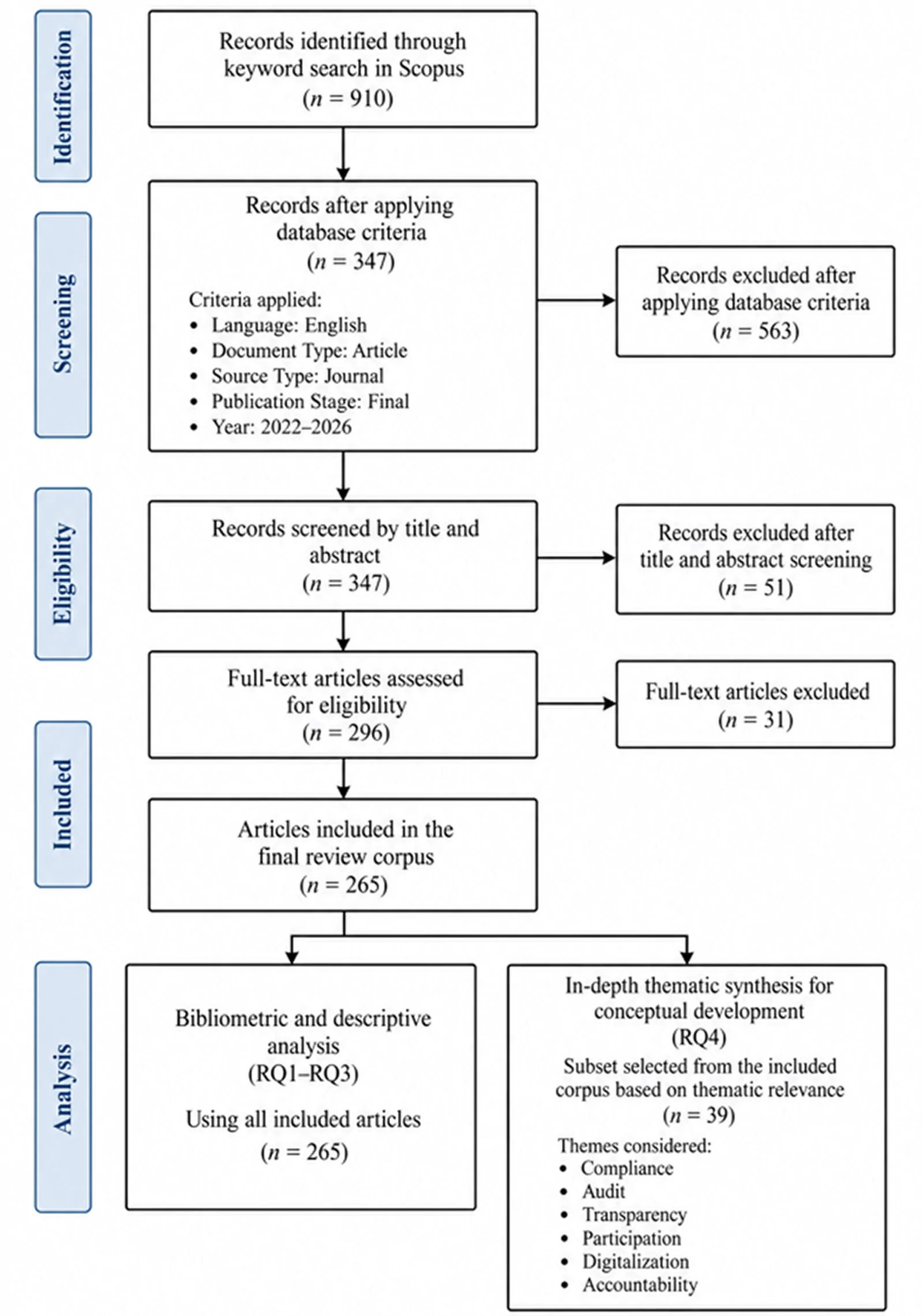
PRISMA flow diagram of literature selection for bibliometric analysis and in-depth thematic synthesis.

The search initially identified 910 records from Scopus. After applying the predefined database criteria, namely English language, article document type, journal source type, final publication stage, and the 2022–2026 publication period, 347 records remained, while 563 records were excluded. Title and abstract screening further reduced the dataset to 296 articles, with 51 records excluded as irrelevant. A further 31 articles were excluded after full-text eligibility assessment. As shown in Figure 1, the final review corpus therefore consisted of 265 articles. These 265 articles were used in the bibliometric and descriptive analyses to address RQ1–RQ3. From this included corpus, a subset of 39 articles was then selected on the basis of thematic relevance for in-depth full-text thematic synthesis to address RQ4, focusing on compliance, audit, transparency, participation, digitalization, and accountability.

## 4. Results

### 4.1 Descriptive overview and publication growth

The annual publication pattern was examined to assess the development of scholarly attention to public financial governance during the review period. As shown in Figure 2, publication output varied across years but displayed a clear increase toward the end of the period.

**Figure 2.**
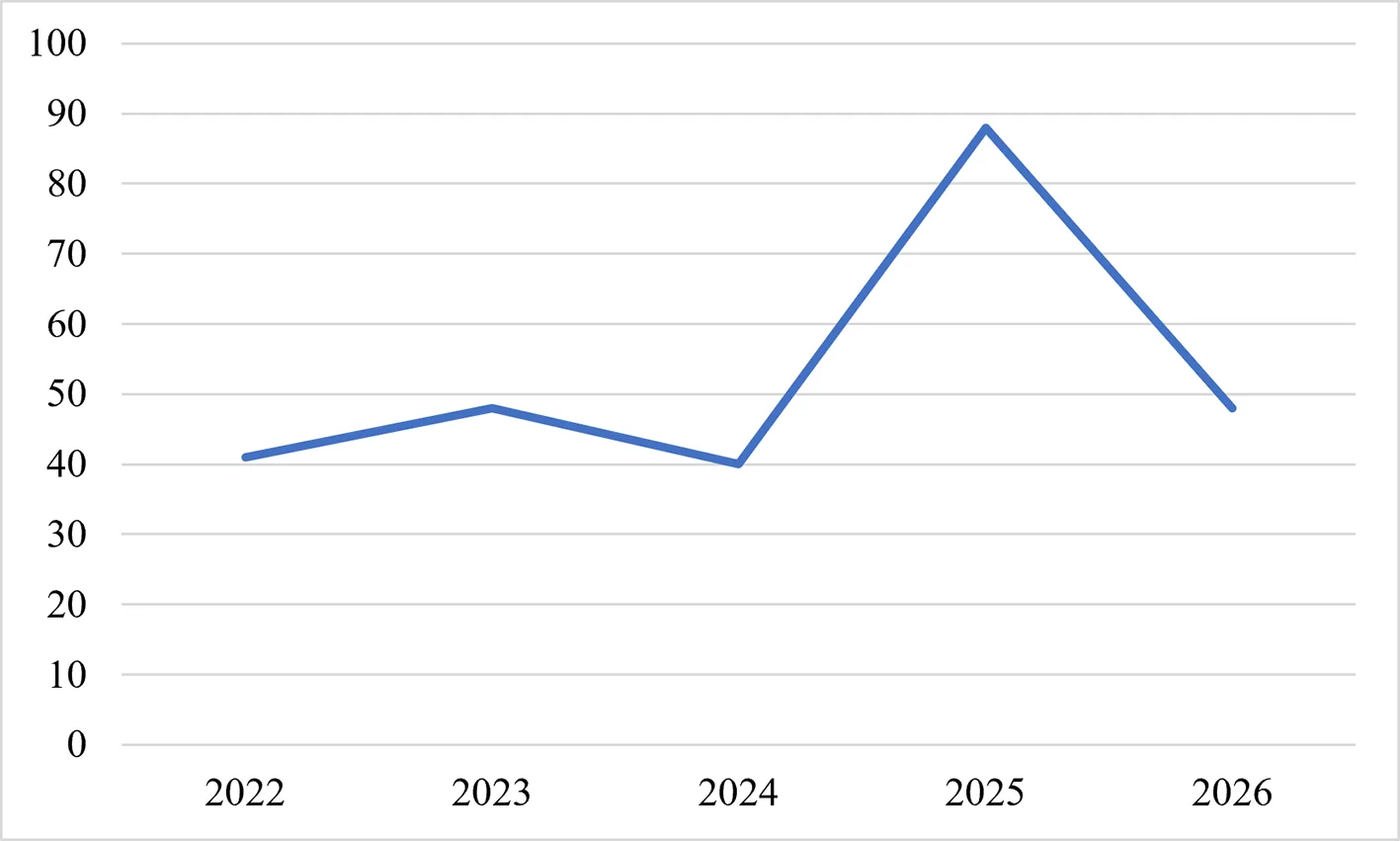
Annual scientific production.

Publication output increased from 41 articles in 2022 to 48 in 2023, before declining slightly to 40 in 2024. It then rose sharply to 88 articles in 2025, marking the highest level of productivity. The decrease to 48 articles in 2026 should be interpreted cautiously because the year may still be incomplete at the time of data collection. Overall, the pattern indicates growing scholarly interest, particularly from 2025 onward. Additional characteristics of the dataset are summarized in Table 1.

**Table 1.** Main data information.

No.	Category	Indicator	Result
1	**Main Data Information**	Timespan	2022–2026
2		Sources (Journals)	179
3		Documents	265
4		Annual Growth Rate (%)	4.02
5		Average Document Age	1.75
6		Average Citations per Document	5.083
7		References	13,385
8	**Document Contents**	Keywords Plus (ID)	347
9		Author’s Keywords (DE)	980
10	**Authors**	Authors	651
11		Authors of Single-authored Documents	43
12	**Author Collaboration**	Single-authored Documents	44
13		Co-authors per Document	2.78
14		International Co-authorships (%)	24.15
15	**Document Types**	Article	265

As shown in Table 1, the dataset comprises 265 journal articles published across 179 sources between 2022 and 2026, indicating a recent and diverse research field. An annual growth rate of 4.02% and an average document age of 1.75 years confirm increasing scholarly attention and the dominance of recent studies. Average citations remain moderate at 5.083 per document, while 13,385 references demonstrate strong engagement with prior literature. The presence of 347 Keywords Plus and 980 author keywords reflects substantial thematic diversity. Collaboration is evident from 651 authors, 44 single-authored documents, 2.78 co-authors per article, and a 24.15% international co-authorship rate, although cross-country collaboration remains moderate.

### 4.2 Influential authors, sources, countries, and documents

This subsection examines the principal intellectual contributors and publication characteristics shaping research on public financial governance. The distribution of the most productive authors is first presented in Figure 3, followed by a broader comparison of influential authors, publication sources, countries, and documents in Table 2.

**Figure 3.**
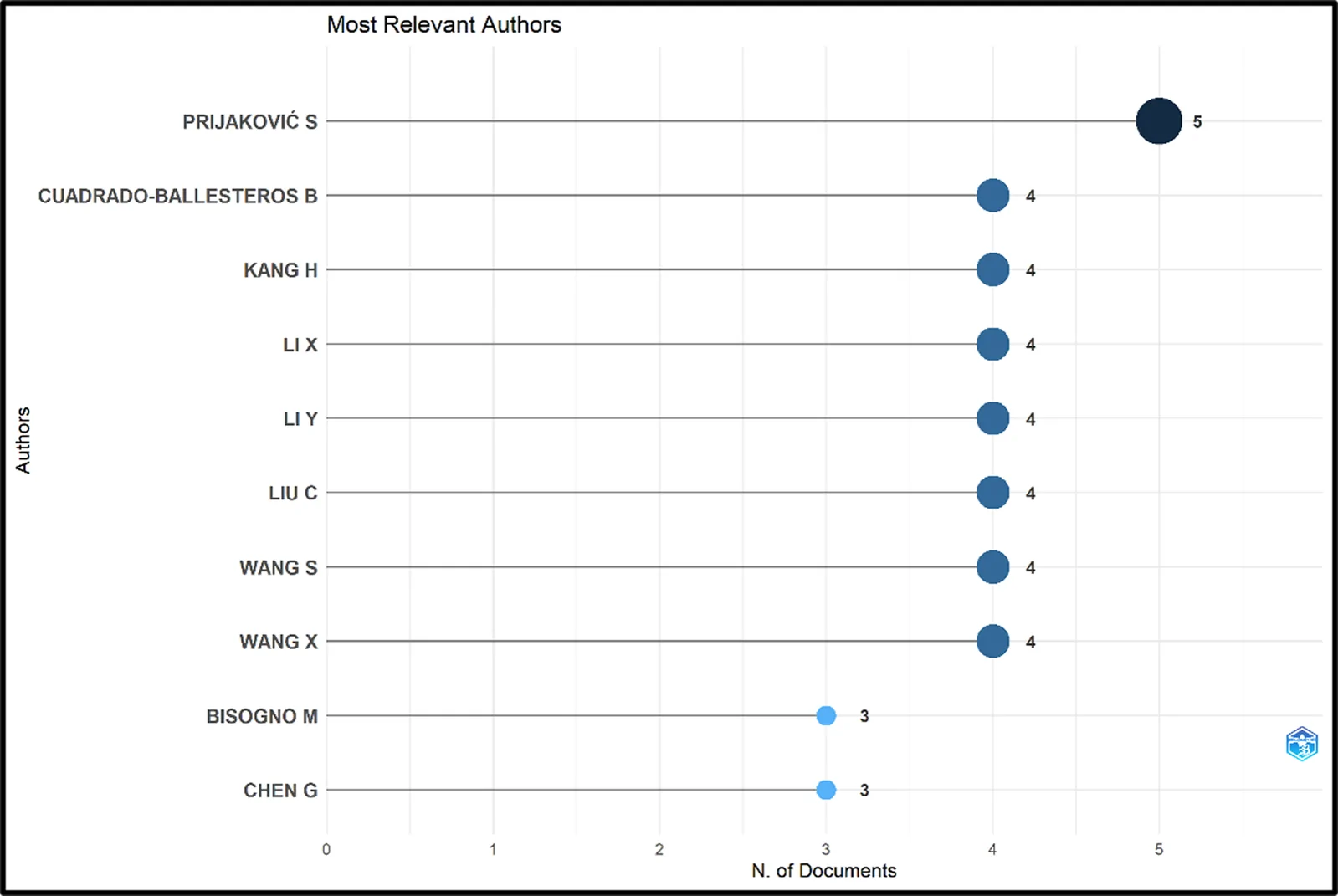
Most relevant author.

**Table 2.** Most influential authors, sources, countries, and documents.

Rank	Most Relevant Authors (N. of Documents)	Most Relevant Sources (N. of Documents)	Most Cited Countries (N. of Citations)	Most Global Cited Documents (Global Citations)
1	Prijaković S (5)	Public Money and Management (13)	China (443)	Chen & Ganapati, 2023, International Review of Administrative Sciences (47)
2	Cuadrado-Ballesteros B (4)	Sustainability (Switzerland) (10)	USA (97)	Gootjes & de Haan, 2022, European Journal of Political Economy (42)
3	Kang H (4)	Journal of Public Budgeting, Accounting and Financial Management (9)	United Kingdom (73)	Cuadrado‐Ballesteros & Bisogno, 2022, Journal of Public Budgeting, Accounting and Financial Management (40)
4	Li X (4)	Applied Economics (4)	Netherlands (56)	Chen et al., 2024, International Review of Financial Analysis (39)
5	Li Y (4)	International Review of Administrative Sciences (4)	Italy (53)	Lino et al., 2022, Financial Accountability and Management (39)
6	Liu C (4)	International Review of Economics and Finance (4)	Georgia (47)	Zhu & Yu, 2024, Applied Economics (30)
7	Wang S (4)	Multidisciplinary Science Journal (4)	South Africa (33)	Li M, 2024, International Review of Financial Analysis (29)
8	Wang X (4)	OIDA International Journal of Sustainable Development (4)	Canada (31)	Sinervo et al., 2024, Journal of Public Budgeting, Accounting and Financial Management (27)
9	Bisogno M (3)	Public Sector Economics (4)	Spain (30)	Ferry et al., 2023, Public Money and Management (27)
10	Chen G (3)	Economic Analysis and Policy (3)	Finland (27)	Bisogno & Cuadrado‐Ballesteros, 2022, Public Management Review (25)

As shown in Figure 3, authorship productivity is relatively dispersed, with no single scholar dominating the field. Prijaković S is the most productive author with five documents, followed by Cuadrado-Ballesteros B, Kang H, Li X, Li Y, Liu C, Wang S, and Wang X with four documents each. Bisogno M and Chen G contributed three documents. This narrow difference among the leading authors indicates a pluralistic knowledge structure supported by several active researchers. To provide a more comprehensive overview of scholarly influence, Table 2 summarizes the leading authors, publication sources, cited countries, and globally cited documents.

As presented in Table 2, scholarly production is distributed among multiple active authors, while publication output is more concentrated in several core journals. Public Money & Management is the leading source with 13 documents, followed by Sustainability with 10 and the Journal of Public Budgeting, Accounting & Financial Management with nine. China records the highest citation influence with 443 citations, substantially exceeding the USA and the United Kingdom. Among the documents, Chen and Ganapati (2023) is the most globally cited with 47 citations, followed by Gootjes & de Haan, (2022) and Cuadrado-Ballesteros & Bisogno, (2022). Overall, Table 2 demonstrates that the field is interdisciplinary, geographically diverse, and supported by several influential authors and recent foundational studies.

### 4.3 Conceptual structure: keyword co-occurrence and thematic map

This subsection examines the conceptual structure of the field by identifying relationships among frequently occurring keywords and assessing the maturity and relevance of major research themes. The keyword network is first presented in Figure 4, followed by the thematic positioning of the identified topics in Figure 5.

**Figure 4.**
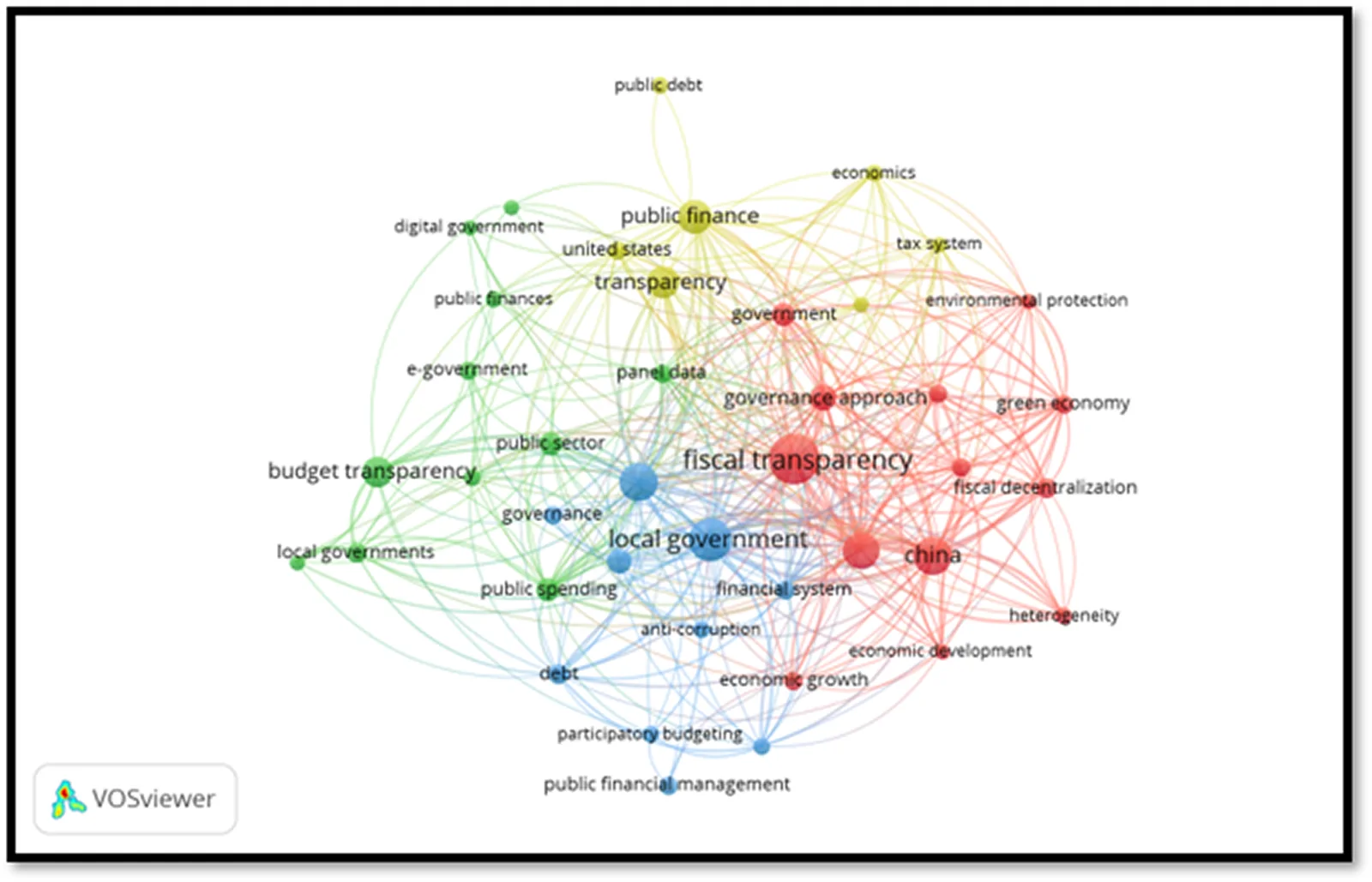
Keyword Co-Occurrence Network.

**Figure 5.**
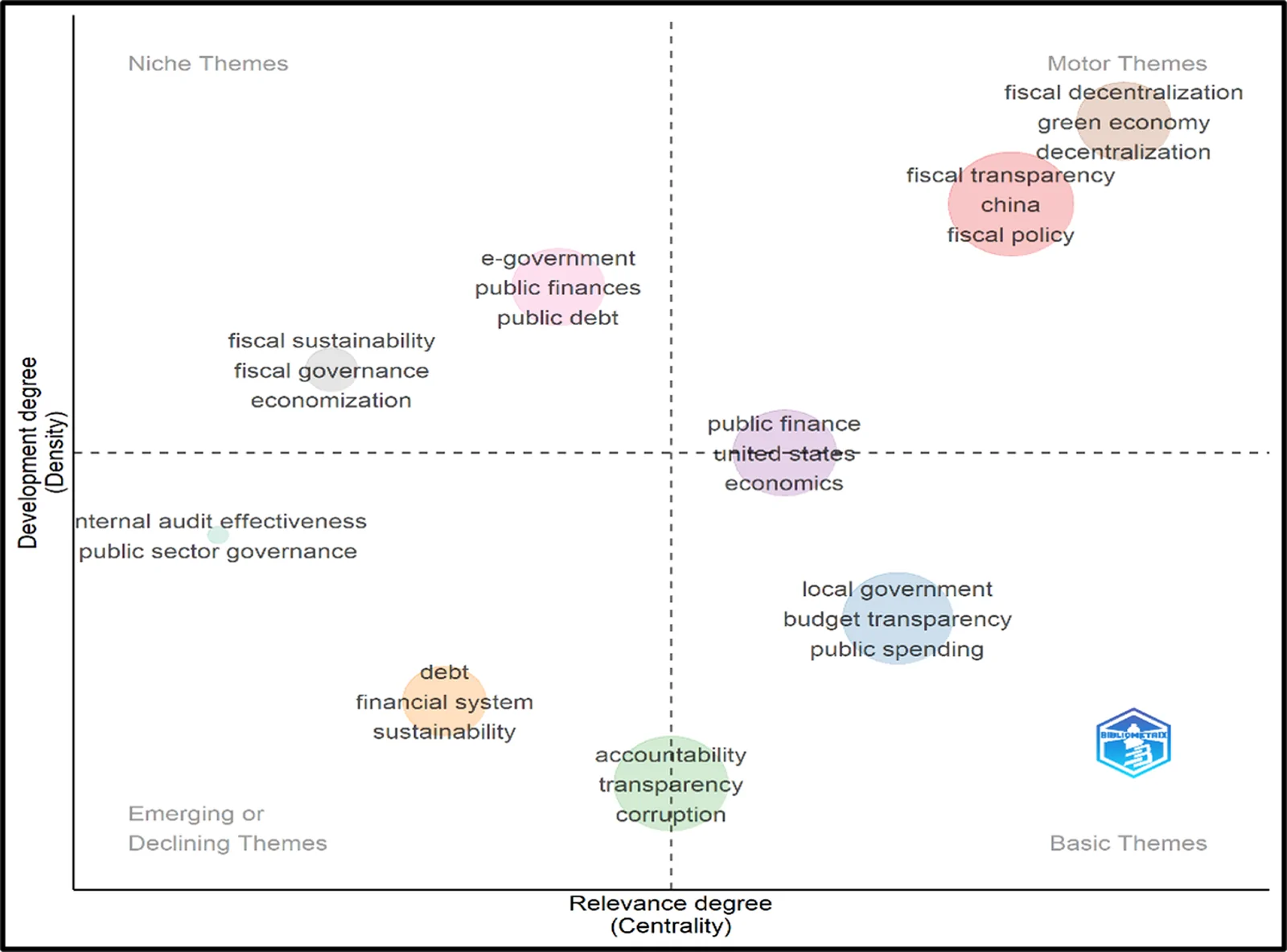
Thematic map.

As shown in Figure 4, the literature is organized around several interconnected themes, with fiscal transparency, local government, accountability, transparency, and public finance forming the central nodes. Fiscal transparency acts as a conceptual bridge linking fiscal policy, decentralization, government, innovation, and economic development. Local government and accountability are connected with corruption, participatory budgeting, public financial management, debt, and sustainability. The network also highlights the importance of digital governance, budget openness, taxation, and public debt. To further assess the development and strategic importance of these themes, their thematic positions are presented in Figure 5.

As illustrated in Figure 5, fiscal transparency, fiscal policy, fiscal decentralization, decentralization, green economy, and China appear as motor themes because they are both highly developed and central to the field. Local government, budget transparency, and public spending constitute basic themes, while accountability, transparency, and corruption remain relevant but require further conceptual development. E-government, public finance, public debt, fiscal sustainability, and fiscal governance appear as specialized themes. Meanwhile, debt, sustainability, internal audit effectiveness, and public-sector governance may represent emerging or declining areas. The main thematic groups and their conceptual meanings are summarized in Table 3.

**Table 3.** Thematic clusters and interpretation.

Cluster	Main Keywords	Interpretation
**Cluster 1** Fiscal transparency and decentralization	fiscal transparency; fiscal policy; fiscal decentralization; decentralization; government; China; green economy; innovation; environmental protection; taxation; tax system	This cluster represents the most mature and central research stream. The thematic map places fiscal transparency, fiscal policy, and fiscal decentralization in the motor-themes quadrant, indicating that fiscal transparency drives current debates on public financial governance. Conceptually, this cluster shows that financial governance is increasingly linked to decentralization, policy reform, environmental agendas, and economic development rather than only technical fiscal reporting.
**Cluster 2** Local government, budget transparency, and public spending	local government; budget transparency; public spending; public sector; local governments; participatory budgeting; public financial management	This cluster reflects the foundational role of local government in public financial governance. The thematic map positions local government, budget transparency, and public spending as basic themes, meaning they are highly relevant to the field but still require deeper theoretical development. The cluster indicates that subnational budgeting, openness of budget information, and public spending accountability form the empirical base for collaborative financial governance.
**Cluster 3** Accountability, transparency, and integrity	accountability; transparency; corruption; anti-corruption; governance; participatory budgeting; public financial management; public value	This cluster shows that accountability and transparency operate as cross-cutting concepts connecting formal financial control with public integrity. Their position near the lower-central area of the thematic map suggests that these themes are highly relevant but still need stronger conceptual integration. In relation to collaborative financial governance, the cluster indicates that financial accountability should not be limited to reporting compliance but should also include anti-corruption, public scrutiny, and multi-actor responsibility.
**Cluster 4** Public finance and macro-fiscal policy	public finance; transparency; economics; public debt; taxation; tax system; United States; economics	This cluster links public financial management with broader macro-fiscal and economic policy debates. The co-occurrence network shows that public finance and transparency bridge fiscal transparency, taxation, public debt, and economics. This indicates that the field is not confined to administrative budgeting, but also examines how fiscal openness relates to taxation, public debt management, economic policy, and national-level fiscal governance.
**Cluster 5** Digital government and open fiscal information	e-government; digital government; public administration; public finances; public debt; public sector; budget transparency	This cluster represents a more specialized research stream around digitalization and open financial information. In the thematic map, e-government, public finances, and public debt appear as niche themes, showing that they are internally developed but less central to the whole field. The cluster is important because it explains how digital platforms, administrative modernization, and access to fiscal data can support transparency and accountability in public financial governance.
**Cluster 6** Financial sustainability, audit, and governance control	debt; financial system; sustainability; fiscal sustainability; fiscal governance; internal audit effectiveness; public sector governance	This cluster captures topics positioned mainly in the emerging or declining themes quadrant. It includes financial sustainability, debt, internal audit effectiveness, and public sector governance, which may represent either new research directions or areas with decreasing attention. For the conceptual agenda of collaborative financial governance, this cluster highlights the need to connect audit, oversight, fiscal sustainability, and governance control with transparency and public accountability mechanisms.

As presented in Table 3, the conceptual structure of the field can be grouped into six major clusters: fiscal transparency and decentralization; local government, budget transparency, and public spending; accountability, transparency, and integrity; public finance and macro-fiscal policy; digital government and open fiscal information; and financial sustainability, audit, and governance control. Together, these clusters demonstrate that public financial governance has evolved beyond technical budgeting and regulatory compliance toward a broader agenda integrating fiscal openness, subnational governance, digitalization, public accountability, participation, and institutional oversight.

### 4.4 Systematic literature review synthesis

To address RQ4, the studies selected for full-text thematic synthesis were examined to identify the governance mechanisms that contribute to the development of collaborative financial governance. The synthesis focused on six recurring dimensions, namely compliance, audit, transparency, participation, digitalization, and accountability. Each study was coded according to whether these dimensions were substantively addressed in its conceptual arguments, empirical findings, or governance implications. As summarized in Table 4, this approach allows the literature to be compared systematically and provides an empirical basis for identifying the dominant and complementary mechanisms that can be integrated into a conceptual agenda for collaborative financial governance. The full synthesis and detailed characteristics of the 39 studies are presented in Appendix 1.

**Table 4.** Thematic synthesis of studies supporting the conceptual agenda for collaborative financial governance.

Study	Compliance	Audit	Transparency	Participation	Digitalization	Accountability
Choudhury & Chutani (2026)	✓		✓		✓	✓
Afadzinu (2026)	✓	✓	✓			✓
Cahyani et al. (2026)	✓		✓	✓	✓	✓
Castañeda-Rodríguez (2022)	✓		✓	✓		✓
Chen & Hu (2025)		✓	✓			✓
Coghill et al. (2026)	✓		✓	✓	✓	✓
Ferry et al. (2023)	✓	✓				✓
Gootjes & de Haan (2022)	✓		✓			✓
Heald & Hodges (2024)	✓	✓	✓			✓
Hoxha et al. (2025)	✓		✓		✓	✓
Mukhamedyarova et al. (2025)	✓	✓	✓	✓		✓
Krynytsia et al. (2024)			✓	✓	✓	✓
Maisyarah (2026)	✓		✓	✓	✓	✓
Mamberti & Minatta (2022)	✓		✓	✓		✓
Mphahlele et al. (2025)	✓	✓	✓		✓	✓
Musiega et al. (2023)	✓	✓	✓	✓		✓
Muzurura & Mutambara (2022)	✓	✓	✓			✓
Ng (2023)	✓	✓	✓			✓
Nonki Tadida (2023)	✓	✓	✓	✓	✓	✓
Poomeesaeng & Srisontisuk (2026)	✓		✓	✓	✓	✓
Saptono & Khozen (2024)	✓		✓	✓		✓
Shaikh et al. (2026)	✓	✓	✓		✓	✓
Siebrits (2025)	✓	✓	✓			✓
Sinervo et al. (2024)			✓	✓	✓	✓
Tóth & Bán-Forgács (2024)	✓		✓	✓		✓
Zheng et al. (2026)	✓	✓	✓	✓		✓
Bronić et al. (2022)	✓		✓		✓	✓
Czekaj et al. (2024)	✓		✓	✓	✓	✓
Wen et al. (2025)			✓	✓	✓	✓
Barisone et al. (2026)	✓	✓	✓	✓	✓	✓
Masoud (2025)	✓	✓	✓		✓	✓
Pham & Dang (2025)	✓		✓	✓		✓
Zarei et al. (2024)	✓		✓			✓
Mahmoud et al. (2025)			✓		✓	✓
Novita (2025)		✓	✓	✓	✓	✓
Witter et al. (2026)	✓	✓	✓	✓	✓	✓
Crowley et al. (2026)	✓					✓
Murray Svidroňová et al. (2023)	✓		✓	✓	✓	✓
Wei et al. (2024)			✓			✓
**Total**	**32**	**17**	**37**	**21**	**20**	**39**

The synthesis presented in Table 4 shows that accountability is the most consistently represented dimension across the selected studies, followed by transparency and compliance, indicating that these concepts form the central foundation of the literature on public financial governance. Audit, participation, and digitalization appear more selectively, but they perform important complementary roles by strengthening oversight, expanding stakeholder involvement, and enabling access to financial information and governance processes. The pattern also suggests that these dimensions should not be understood as isolated or strictly sequential components. Rather, the studies demonstrate that formal compliance and audit mechanisms require transparency and institutional capacity, transparency becomes more meaningful when accompanied by participation and accessible information, and digitalization can facilitate these relationships when supported by appropriate safeguards and accountability arrangements. Accordingly, Table 4 provides the basis for conceptualizing collaborative financial governance as an integrated and multi-actor governance architecture rather than a collection of independent mechanisms.

## 5. Discussion

### 5.1 From compliance-based to collaborative financial governance

The findings indicate that public financial governance is moving beyond a narrow compliance-based orientation toward a more open and collaborative model. Gootjes and de Haan (2022) show that fiscal rules remain important for budget discipline, but their effectiveness depends on adequate transparency. Compliance with regulations, reporting procedures, and audit requirements remains the institutional foundation of public financial management. Boufounou et al. (2024) add that effective local-government internal control depends on regulatory compliance, competent personnel, collaboration, and information systems. Nevertheless, substantive accountability also requires accessible information and stakeholder interaction ( Coghill et al., 2026; Lino et al., 2022).

Audit and oversight remain indispensable within a broader accountability system. Ferry et al. (2023) emphasize the public-interest role of public-sector auditing, while Mukhamedyarova et al. (2025) show that audit institutions can strengthen transparency and local budget governance. Auditing also contributes to fraud prevention and corruption control ( Branet & Hategan, 2024). However, findings generate limited accountability when disconnected from disclosure, legislative scrutiny, institutional follow-up, or citizen monitoring. Lino et al. (2023) show that audit digitalization can influence local budget planning, while machine learning may improve audit-outcome interpretation ( Mabelane et al., 2023). National audit is more effective against corruption when combined with public participation and good governance ( Su et al., 2023).

Overall, compliance remains necessary but insufficient for collaborative financial governance. Compliance can facilitate collaboration when formal rules clarify institutional responsibilities, establish minimum standards for transparency and accountability, protect oversight functions, and provide legitimate procedures through which multiple actors can access information and participate in financial governance. For example, fiscal rules are more effective when accompanied by adequate budget transparency, suggesting that formal control can support broader accountability when it is connected to openness and scrutiny ( Gootjes & de Haan, 2022).

However, compliance becomes proceduralism when adherence to reporting requirements, deadlines, standardized procedures, or digital systems becomes an end in itself and is disconnected from substantive organizational change, information usability, stakeholder participation, and institutional follow-up ( Choudhury & Chutani, 2026; Lino et al., 2022). Formal adoption alone may therefore create the appearance of accountability without strengthening actual transparency or public engagement ( Castañeda-Rodríguez, 2022). Accordingly, collaborative financial governance does not replace compliance but repositions it as an institutional foundation that should remain sufficiently flexible and connected to transparency, participation, oversight, and responsiveness. Participatory budgeting and collaborative planning can further strengthen scrutiny and resource allocation ( Sousa, 2024; Zheng et al., 2026), while citizen participation supports more responsive public-service governance ( Torres-Sandoval et al., 2025).

### 5.2 Multi-actor accountability as the core of collaborative financial governance

Collaborative financial governance is grounded in multi-actor accountability. Ferry et al. (2023) show that public financial accountability involves audit institutions, regulators, public managers, elected officials, and other actors across the accountability system. It therefore extends beyond vertical agency–auditor relationships to include horizontal accountability among state institutions and social accountability through citizens, civil society, taxpayers, media, and digital communities ( van den Boogaard et al., 2022).

Each actor performs a distinct function. Legislatures authorize budgets, audit institutions assess legality and performance, civil society and the media interpret financial information, and citizens contribute knowledge about service outcomes. Digital platforms connect disclosure with public feedback. Taxpayer engagement and fiscal transparency can strengthen accountability by linking revenue collection with government responsiveness, while collaboration depends on interaction among actors with different mandates, information, and legitimacy ( Sinervo et al., 2024; van den Boogaard et al., 2022).

Local government illustrates this accountability ecosystem. Participatory budgeting enables citizens to influence financial decisions and links resource allocation with sustainable governance ( Sinervo et al., 2024). Audit outcomes support the evaluation of local financial management, while digital monitoring can strengthen budgetary control ( Kenetey & Popesko, 2025; Mabelane et al., 2023). Local financial governance becomes collaborative when formal institutions, citizens, and digital mechanisms operate together.

However, multiple actors do not automatically ensure accountability. Fragmented responsibilities, overlapping mandates, unequal information access, and symbolic participation may weaken outcomes. Sumiyana et al. (2023) show that political influence can undermine auditor independence and audit quality, while participation may become ceremonial when citizen input is disconnected from decisions or budget follow-up. Clear institutional roles, accessible information, procedural safeguards, and links between public input and official responses are therefore essential ( Sumiyana et al., 2023).

Multi-actor accountability should therefore be understood as an organized architecture integrating vertical, horizontal, and social accountability. Wang and Sun (2026) indicate that social-media participation may support municipal fiscal health when combined with fiscal transparency. Such interaction transforms financial information and oversight findings into shared processes of questioning, explanation, correction, and institutional learning ( Wang & Sun, 2026; Xiao & Wang, 2024).

### 5.3 Transparency, oversight, and participation as bridging themes

Transparency, oversight, and participation connect formal compliance with collaborative financial governance. Chen and Ganapati (2023) explain that transparency can support corruption control when financial information is publicly available and reinforced by accountability mechanisms. By reducing information asymmetry, transparency enables external actors to examine budgets, revenues, expenditures, audit findings, and fiscal risks. However, disclosure has limited value when information is delayed, incomplete, inaccessible, or overly technical. Transparency becomes substantive only when users can understand and apply financial information to evaluate government decisions ( Bisogno & Cuadrado-Ballesteros, 2022; Chen & Ganapati, 2023).

Oversight provides the institutional capacity to verify financial information, identify irregularities, evaluate performance, and recommend corrective action. Ferry et al. (2023) position audit and oversight within a regulatory space shaped by independence, mandate, inspection authority, and public-interest responsibility. Their contribution is strongest when audit findings are publicly communicated, followed up by responsible agencies, and scrutinized by legislatures and citizens. Digital audit systems may also support budget planning and administrative learning rather than functioning only as retrospective control ( Ferry et al., 2023; Lino et al., 2023).

Participation adds a social accountability dimension. Bisogno and Cuadrado-Ballesteros (2022) show that e-government and e-participation may broaden access to budgeting processes and improve budget transparency. Participatory budgeting also enables citizens to influence resource allocation and public priorities, rather than merely monitor completed expenditure ( Sinervo et al., 2024). Thus, participation transforms citizens from passive recipients of fiscal information into active financial-governance actors ( Bisogno & Cuadrado-Ballesteros, 2022; Sinervo et al., 2024).

These mechanisms are mutually reinforcing: transparency provides information, oversight evaluates it, and participation enables public scrutiny. Xiao and Wang (2024) emphasize that fiscal transparency requires legislative involvement, audit institutions, established procedures, and citizen participation. Consequently, transparency without oversight may become symbolic, oversight without transparency may remain closed, and participation without reliable information may lack influence ( Chen & Ganapati, 2023; Xiao & Wang, 2024).

Digital platforms further integrate disclosure, monitoring, and participation. Public-data platforms can expand fiscal transparency, while blockchain monitoring may strengthen local budgetary control ( Han et al., 2025; Kenetey & Popesko, 2025). However, digital openness contributes to collaborative governance only when supported by data quality, accessibility, user capacity, institutional responsiveness, and meaningful follow-up.

### 5.4 Conceptual agenda for collaborative financial governance

The synthesis indicates that collaborative financial governance should first be understood as an institutional architecture in which formal control provides necessary safeguards without becoming the final objective of governance. Gootjes and de Haan (2022) demonstrate that fiscal rules become more effective when supported by budget transparency, while formal standards and controls may produce limited substantive outcomes when they are disconnected from organizational capacity, institutional context, or meaningful implementation ( Choudhury & Chutani, 2026; Castañeda-Rodríguez, 2022; Heald & Hodges, 2024; Zarei et al., 2024). Audit therefore complements compliance by examining legality, performance, and responsibility, but its contribution depends on independence, institutional authority, follow-up, and protection from political interference ( Afadzinu, 2026; Ferry et al., 2023; Mukhamedyarova et al., 2025; Muzurura & Mutambara, 2022; Ng, 2023). Nonki Tadida (2023) further shows that effective oversight increasingly requires cooperation among audit institutions, legislatures, civil society, media, and other accountability actors, a pattern also reflected in multi-institutional oversight arrangements ( Mphahlele et al., 2025; Musiega et al., 2023; Siebrits, 2025).

Transparency subsequently becomes a connecting mechanism because financial information enables actors outside administrative hierarchies to evaluate and question fiscal decisions. However, disclosure alone is insufficient when information is inaccessible, difficult to interpret, or disconnected from institutional response ( Bronić et al., 2022; Coghill et al., 2026; Czekaj et al., 2024; Tóth & Bán-Forgács, 2024). Wei et al. (2024) show that fiscal transparency shapes citizens’ assessments of government fiscal performance and responsibility, while national auditing can strengthen fiscal openness and accountability ( Chen & Hu, 2025). Participation extends this logic by transforming transparency into interaction through participatory budgeting, public scrutiny, stakeholder engagement, and citizen influence over resource allocation ( Mamberti & Minatta, 2022; Saptono & Khozen, 2024; Sinervo et al., 2024; Zheng et al., 2026). Nevertheless, participation requires political commitment, institutionalization, feedback, and sufficient administrative and civic capacity to avoid symbolic involvement ( Maisyarah, 2026; Murray Svidroňová et al., 2023; Novita, 2025).

Digitalization further links these mechanisms by expanding access, monitoring, traceability, and interaction, but its governance effects remain context-dependent. Mahmoud et al. (2025) demonstrate that e-government does not produce uniform governance outcomes, while digital financial systems can strengthen transparency and accountability when supported by capacity, safeguards, and institutional integration ( Cahyani et al., 2026; Hoxha et al., 2025; Krynytsia et al., 2024; Shaikh et al., 2026; Wen et al., 2025). Collaborative digital infrastructures can additionally support shared governance and auditability across institutional boundaries ( Barisone et al., 2026; Masoud, 2025). Accordingly, digitalization should be treated as an enabling mechanism rather than an independent guarantee of collaborative governance.

Taken together, RQ4 is answered by conceptualizing collaborative financial governance as an interactive, multi-actor accountability architecture rather than a sequential model. Poomeesaeng and Srisontisuk (2026) provide the clearest evidence that fiscal capacity can be co-produced through formal, task-specific, and informal collaborative networks. This architecture must balance local autonomy with central oversight and accountability ( Pham & Dang, 2025; Witter et al., 2026), because excessive upward control can weaken local ownership, coordination, and long-term capacity ( Crowley et al., 2026). Thus, compliance, audit, transparency, participation, digitalization, and accountability operate as mutually reinforcing mechanisms whose effectiveness depends on institutional capacity, responsiveness, trust, clear responsibilities, and cross-actor coordination.

Building on the thematic synthesis of the 39 studies, the findings suggest that collaborative financial governance is better understood as an integrated governance architecture rather than as a causal sequence among variables. The literature consistently highlights six recurring domains, namely compliance, audit, transparency, participation, digitalization, and accountability, but it does not provide sufficient evidence to establish a fixed directional relationship among them. Instead, these domains appear as complementary governance mechanisms whose relevance depends on institutional and contextual conditions, including political will, institutional capacity, regulatory support, resources, technology, trust, and collaborative culture. Accordingly, Figure 6 translates the synthesis into a conceptual agenda that organizes these recurring domains around collaborative financial governance while explicitly avoiding the assumption that one dimension necessarily causes another.

**Figure 6.**
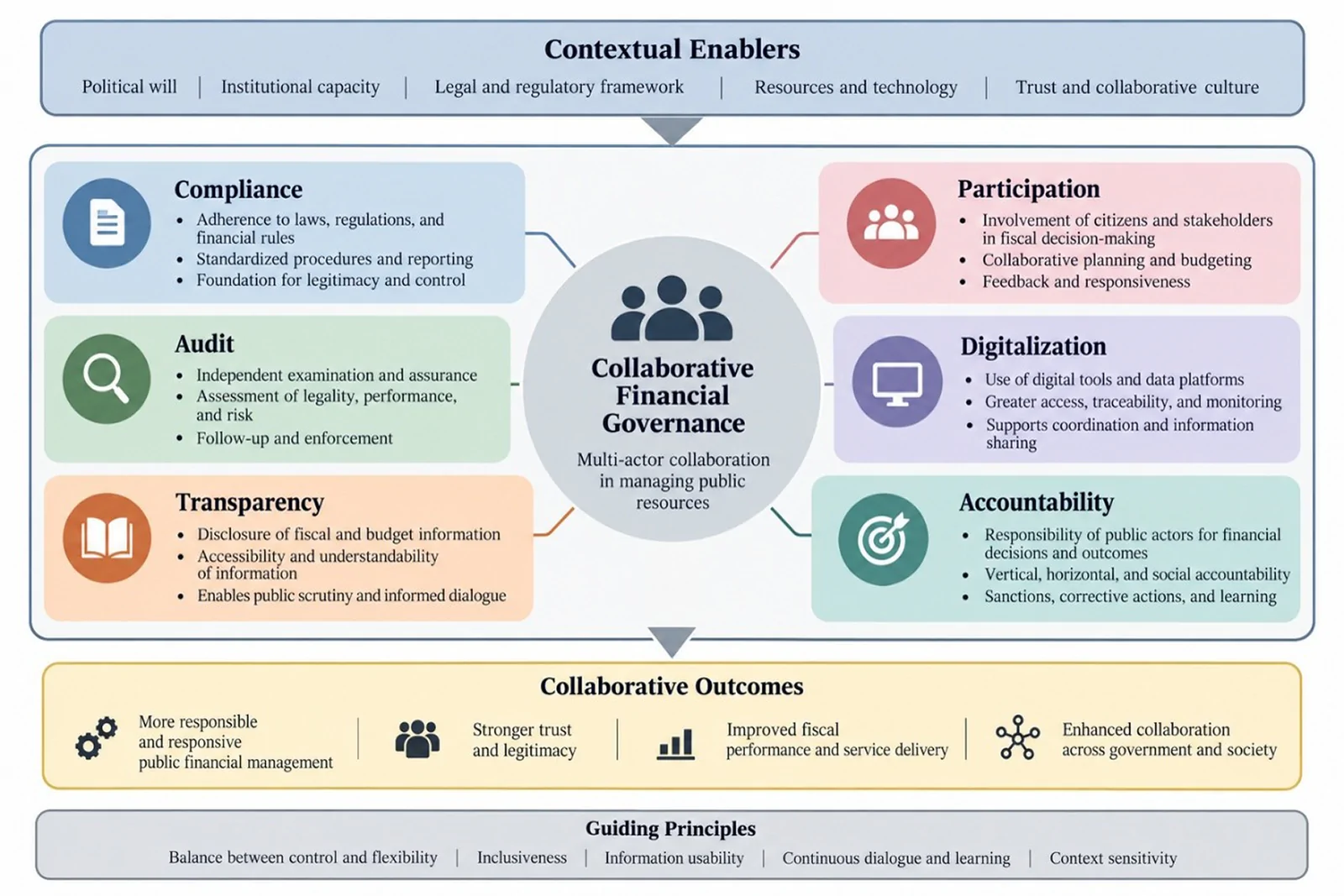
Conceptual Agenda for Collaborative Financial Governance.

As shown in Figure 6, collaborative financial governance is positioned at the center of a broader governance configuration in which compliance, audit, transparency, participation, digitalization, and accountability represent complementary domains rather than independent or sequential variables. Compliance provides the formal and regulatory foundation, while audit contributes assurance and oversight; transparency improves the availability and usability of fiscal information; participation creates opportunities for citizens and stakeholders to engage in financial decision-making; digitalization facilitates access, monitoring, traceability, and coordination; and accountability links financial decisions with responsibility, scrutiny, and corrective action. However, the literature also indicates that these mechanisms are effective only when supported by enabling conditions and guiding principles such as institutional capacity, political commitment, inclusiveness, information usability, balanced control and flexibility, continuous dialogue, and contextual sensitivity ( Murray Svidroňová et al., 2023; Nonki Tadida, 2023; Poomeesaeng & Srisontisuk, 2026; Witter et al., 2026). Therefore, the framework should be interpreted as a conceptual organizing structure derived from the literature, rather than as an empirically tested causal model, and it provides a basis for future research to examine how these governance domains interact across different public financial management settings.

## 6. Implications

### 6.1 Theoretical implications

This study extends the theoretical understanding of public financial management by conceptualizing collaborative financial governance as an integrated governance architecture rather than a sequential causal model. Conventional public financial management emphasizes regulatory compliance, accounting, reporting, and formal control, but the findings indicate that these mechanisms represent only part of a broader governance configuration. Fiscal rules remain important, yet their effectiveness is strengthened when financial information is sufficiently transparent and accessible ( Gootjes & de Haan, 2022). Accordingly, the proposed framework brings together six complementary domains, namely compliance, audit, transparency, participation, digitalization, and accountability, without assuming that one domain necessarily precedes or causes another.

The framework also broadens the conceptualization of accountability. Public-sector audit contributes through independence, regulatory authority, inspection, and performance assessment ( Ferry et al., 2023), while financial accountability also operates horizontally across public institutions and socially through citizens, taxpayers, civil society, media, and other stakeholders ( van den Boogaard et al., 2022). Transparency, participation, and digitalization therefore function as complementary governance mechanisms that can widen access to information, public scrutiny, and stakeholder interaction ( Chen & Ganapati, 2023; Han et al., 2025; Sinervo et al., 2024). The theoretical contribution of this study thus lies in positioning collaborative financial governance as a multi-actor configuration of formal control, institutional oversight, fiscal openness, stakeholder engagement, digital support, and accountability, whose operation is shaped by contextual conditions such as institutional capacity, political commitment, trust, responsiveness, and coordination.

### 6.2 Methodological implications

Methodologically, this study demonstrates the value of combining bibliometric analysis with systematic thematic synthesis to move from descriptive mapping toward conceptual interpretation. Bibliometric analysis was used to examine the 265-article corpus through publication trends, influential contributors, citation structures, keyword relationships, and thematic positioning ( Donthu et al., 2021). However, keyword co-occurrence and thematic maps identify patterns of association rather than explaining the substantive meaning of those relationships. For this reason, systematic interpretation remained necessary to understand how the themes identified bibliometrically were discussed within the underlying literature ( Zupic & Čater, 2015).

The thematic synthesis provides a methodological bridge between science mapping and conceptual development. By coding the studies relevant to RQ4 according to compliance, audit, transparency, participation, digitalization, and accountability, the study translated bibliometric patterns into an organizing framework grounded in substantive evidence. This approach is particularly appropriate because the reviewed literature is heterogeneous in research design and does not provide a consistent basis for specifying causal relationships among the six domains. Consequently, the resulting framework is presented as a conceptual synthesis rather than an empirically tested structural model. More broadly, the study reinforces the importance of transparent screening, consistent metadata preparation, keyword standardization, and explicit alignment between bibliometric outputs, thematic interpretation, and research questions ( Aria & Cuccurullo, 2017; Mukherjee et al., 2022; Page et al., 2021).

### 6.3 Practical implications

Practically, the findings suggest that governments should evaluate public financial governance as an interconnected system rather than improving individual mechanisms in isolation. Reliable accounting, reporting, compliance, and audit remain necessary, but these mechanisms should be accompanied by financial information that is accessible and understandable, meaningful stakeholder participation, effective feedback channels, and digital systems that support monitoring and information sharing. Technical disclosure alone is insufficient when citizens and other stakeholders cannot interpret or use financial information to evaluate government decisions ( Chen & Ganapati, 2023; Gourfinkel, 2022).

Government agencies should therefore strengthen the usability of budget plans, expenditures, fiscal risks, debt information, audit findings, and corrective actions through citizen-oriented reporting, open-data platforms, and appropriate digital tools. Digitalization should be treated as an enabling mechanism rather than merely as administrative automation, because its contribution depends on data quality, institutional integration, user capacity, and follow-up ( Han et al., 2025; Lino et al., 2022, 2023). Likewise, participation should extend beyond formal consultation by providing opportunities for stakeholders to contribute to priority setting, budgeting, monitoring, and evaluation, accompanied by clear institutional responses to public input ( Sinervo et al., 2024). In practice, collaborative financial governance therefore requires a balance between control and flexibility, institutional authority and stakeholder involvement, and transparency and information usability.

### 6.4 Policy implications

The policy implication of this study is that public financial management reform should be designed as a coordinated governance architecture, not as a linear sequence of separate reforms. Policymakers should maintain formal compliance and audit safeguards while simultaneously creating conditions that support transparency, participation, digital access, and accountability. Fiscal rules and formal controls remain essential, but their governance value depends on the wider institutional environment in which they operate ( Gootjes & de Haan, 2022). Similarly, audit policy should protect institutional independence, professional capacity, access to information, and effective follow-up, particularly because political interference may weaken substantive oversight ( Ferry et al., 2023; Sumiyana et al., 2023).

Policy design should also recognize the contextual enablers identified in the conceptual agenda, including political commitment, institutional capacity, regulatory support, resources and technology, trust, and collaborative culture. Transparency policies should emphasize not only disclosure but also timeliness, accessibility, comprehensibility, and usability, while participation policies should provide institutionalized channels for consultation, community proposals, monitoring, and official feedback ( Shybalkina & Nabatchi, 2024; Xiao & Wang, 2024). Digital policies should support open data, audit trails, interoperability, monitoring, and two-way communication rather than merely digitizing existing procedures ( Han et al., 2025). Accordingly, public financial governance policy should be guided by inclusiveness, balanced control and flexibility, information usability, continuous dialogue and learning, and sensitivity to institutional context, enabling the six governance domains to operate coherently within different public-sector environments.

## 7. Conclusions

This study mapped the development and conceptual structure of public financial governance research from 2022 to 2026 and integrated the bibliometric and systematic review findings into a conceptual agenda for collaborative financial governance. The bibliometric evidence indicates a generally increasing trajectory of scholarly attention, with publication output reaching its highest level in 2025. Research influence is distributed across multiple contributors rather than concentrated in a single scholar, while Public Money & Management emerged as the leading publication source, China recorded the highest citation impact, and Chen and Ganapati (2023) was the most globally cited document. The intellectual structure of the field is centered on fiscal transparency, fiscal policy, and fiscal decentralization, complemented by research on local government, budget transparency, public spending, accountability, participation, digital government, audit, and fiscal sustainability.

The systematic synthesis advances this literature by conceptualizing collaborative financial governance as an integrated governance architecture rather than a sequential causal model. Six interrelated domains—compliance, audit, transparency, participation, digitalization, and accountability—emerge as complementary mechanisms through which public financial management can move beyond a predominantly compliance-based orientation toward broader multi-actor governance. Their effectiveness, however, is contingent on institutional and contextual conditions, including political commitment, regulatory support, institutional capacity, trust, information usability, responsiveness, and coordination among governmental and non-governmental actors. The contribution of this study therefore lies not only in mapping the field, but also in providing a conceptual structure that connects formal financial control with openness, participation, digital support, and distributed accountability.

Several limitations should be acknowledged. First, the 2026 publication data were incomplete at the time of data collection, so trends for the final year remain provisional. Second, bibliometric networks identify patterns of intellectual association but cannot establish causal relationships among governance dimensions. Third, the proposed framework is derived from thematic synthesis across heterogeneous institutional and national contexts and has not yet been empirically validated. Future research should update the bibliometric structure after the complete 2026 publication cycle, test the proposed framework through comparative, qualitative, quantitative, or mixed-method studies, and examine how contextual conditions shape the interaction among governance domains across different public financial management settings.

## Ethics Statement

Ethical approval and consent were not required.

## Data Availability

Figshare. Data Systematic Literature Review Collaborative Financial Governance. https://doi.org/10.6084/m9.figshare.33582361 ( Saimima et al., 2026). This project contains the following data:
• Appendix 1 (Summary of Studies Included in RQ4) • PRISMA 2020 Checklist.pdf • Data Bibliometric.xlsx Data are available under the terms of the Creative Commons Attribution 4.0 International license (CC-BY 4.0). Figshare: PRISMA checklist for “From Compliance-Based to Collaborative Financial Governance: A Bibliometric-Systematic Review of Multi-Actor Accountability in Public Financial Management”. https://doi.org/10.6084/m9.figshare.33582361 ( Saimima et al., 2026). Data are available under the terms of the Creative Commons Attribution 4.0 International license (CC-BY 4.0).
