# Supplementary material for: From Compliance-Based to Collaborative Financial Governance: A Bibliometric-Systematic Review of Multi-Actor Accountability in Public Financial Management
Source: F1000Res. 2026 Nov 2;15:1701. Originally published 2026 Oct 12. [Version 2] doi: 10.12688/f1000research.187613.2 (PMC13740455; doi:10.12688/f1000research.187613.2)
Supplement: Peer review history

# Reviewer report: Reviewer response for version 2

**DOI:** 10.5256/f1000research.213593.r536993

**Published:** 2026-11-05

**Authors:** Muhammad Khairul Anwar

**Affiliations:** 1 State University of Surabaya, Surabaya, Indonesia

The current version of the manuscript is excellent. I commend the author for revising it so well, and therefore I recommend that it be approved.

Are the rationale for, and objectives of, the Systematic Review clearly stated?

Yes

Is the statistical analysis and its interpretation appropriate?

Not applicable

If this is a Living Systematic Review, is the ‘living’ method appropriate and is the search schedule clearly defined and justified? (‘Living Systematic Review’ or a variation of this term should be included in the title.)

Not applicable

Are sufficient details of the methods and analysis provided to allow replication by others?

Partly

Are the conclusions drawn adequately supported by the results presented in the review?

Yes

Reviewer Expertise:

Public Administration, Policy, and Governance

I confirm that I have read this submission and believe that I have an appropriate level of expertise to confirm that it is of an acceptable scientific standard.

# Reviewer report: Reviewer response for version 2

**DOI:** 10.5256/f1000research.213593.r536992

**Published:** 2026-11-04

**Authors:** Eka Desy Purnama

**Affiliations:** 1 Universitas Kristen Krida Wacana, Jakarta, Jakarta, Indonesia

The author has revised the manuscript based on my recommendations.

Are the rationale for, and objectives of, the Systematic Review clearly stated?

Yes

Is the statistical analysis and its interpretation appropriate?

Not applicable

If this is a Living Systematic Review, is the ‘living’ method appropriate and is the search schedule clearly defined and justified? (‘Living Systematic Review’ or a variation of this term should be included in the title.)

Not applicable

Are sufficient details of the methods and analysis provided to allow replication by others?

Partly

Are the conclusions drawn adequately supported by the results presented in the review?

Yes

Reviewer Expertise:

Human Resource Management, Financial Management, Operations Management, Marketing Management, Knowledge Management, and Applied Economics.

I confirm that I have read this submission and believe that I have an appropriate level of expertise to confirm that it is of an acceptable scientific standard.

# Reviewer report: Reviewer response for version 1

**DOI:** 10.5256/f1000research.207066.r529690

**Published:** 2026-10-16

**Authors:** Eka Desy Purnama

**Affiliations:** 1 Universitas Kristen Krida Wacana, Jakarta, Jakarta, Indonesia

**Bibliometric Findings**

 The bibliometric analysis provides a valuable overview of research developments in public financial management. Nevertheless, two aspects require further refinement.

1. The publication trends presented in Figure 2 should be interpreted with greater consideration of the incomplete 2026 dataset, as the literature search was conducted in July. This temporal limitation should be reflected more explicitly in the discussion of research productivity.
2. The interpretation of Figure 5 requires greater precision. Themes classified as emerging or declining should be discussed cautiously, as their positioning within the thematic map does not necessarily indicate a definitive increase or decrease in scholarly attention.

**Methodological Transparency and Analytical Consistency**

 The integration of bibliometric analysis and thematic synthesis is appropriate for the research objectives. Certain methodological procedures require additional clarification to improve the transparency of the review.

1. Section 3.7 should specify how conceptually related keywords, particularly fiscal transparency and budget transparency, were standardized. Clarifying whether these terms were merged during bibliometric analysis or grouped solely for thematic interpretation would improve analytical transparency.
2. Further explanation is also needed regarding the selection of 39 studies from the initial 265 articles. The authors should clarify the criteria used to determine their thematic relevance and contribution to the conceptual synthesis.

**Theoretical and Practical Implications**

1. The framework presented in Figure 6 offers a meaningful integration of key financial governance dimensions. Its conceptual contribution would be strengthened by consistently distinguishing relationships inferred from the literature from those established through empirical validation.
2. The practical and policy implications in Sections 6.3 and 6.4 could also be strengthened by incorporating a relevant example from the reviewed studies. Illustrating how transparency, institutional oversight, and stakeholder participation operate as complementary mechanisms would enhance the practical applicability of the proposed framework.

**Recommendation**

 The manuscript makes a relevant contribution to public financial management and collaborative governance research. The issues identified primarily concern analytical precision, methodological clarification, and the presentation of conceptual implications. These matters can be adequately addressed through minor revisions to improve the overall clarity and quality of the manuscript.

Are the rationale for, and objectives of, the Systematic Review clearly stated?

Yes

Is the statistical analysis and its interpretation appropriate?

Not applicable

If this is a Living Systematic Review, is the ‘living’ method appropriate and is the search schedule clearly defined and justified? (‘Living Systematic Review’ or a variation of this term should be included in the title.)

Not applicable

Are sufficient details of the methods and analysis provided to allow replication by others?

Partly

Are the conclusions drawn adequately supported by the results presented in the review?

Yes

Reviewer Expertise:

Human Resource Management, Financial Management, Operations Management, Marketing Management, Knowledge Management, and Applied Economics.

I confirm that I have read this submission and believe that I have an appropriate level of expertise to confirm that it is of an acceptable scientific standard, however I have significant reservations, as outlined above.

# Response

**Published:** 2026-10-26

**Authors:** Simon Saimima

**Affiliations:** Public Administration, Universitas Brawijaya, Malang, East Java, Indonesia

We thank the reviewer for the constructive comments and recommendations. We have revised the manuscript to address the points raised, as detailed below.

**Bibliometric Findings**

 Comment 1: Clarify the interpretation of publication trends given the incomplete 2026 dataset.

 Response: We have revised Section 4.1 and the caption of Figure 2 to clarify that the 2026 data cover only part of the year. The interpretation has been adjusted to avoid suggesting a decline in research productivity.

 Comment 2: Interpret emerging or declining themes in Figure 5 more cautiously.

 Response: We have revised Section 4.3 and Table 3 to clarify that thematic positioning reflects centrality and density.

**Methodological Transparency and Analytical Consistency**

 Comment 3: Clarify the standardization of related keywords, particularly fiscal transparency and budget transparency.

 Response: We have revised Section 3.7 to clarify that these terms were retained separately in the bibliometric analysis, with their conceptual connections addressed during thematic interpretation.

 Comment 4: Explain the selection criteria for the 39 studies included in the thematic synthesis.

 Response: We have revised Section 3.6 to specify the thematic relevance criteria used to select the 39 studies from the 265 articles, including their contribution to the proposed conceptual framework.

**Theoretical and Practical Implications**

 Comment 5: Distinguish conceptual relationships in Figure 6 from empirically validated findings.

 Response: We have revised Section 5.4 to clarify that the proposed framework is based on thematic synthesis and requires further empirical validation.

 Comment 6: Strengthen the practical and policy implications with an example from the reviewed studies.

 Response: We have added an example from Musiega et al. (2023) to Sections 6.3 and 6.4, illustrating the relevance of budget transparency, institutional oversight, and stakeholder participation in local financial governance.

**Recommendation**

 Comment: The manuscript makes a relevant contribution, and the identified issues can be addressed through minor revisions.

 Response: We appreciate the reviewer's positive assessment and constructive recommendations. The revisions have improved the methodological transparency, analytical precision, and presentation of the manuscript's conceptual and practical contributions.

# Reviewer report: Reviewer response for version 1

**DOI:** 10.5256/f1000research.207066.r529689

**Published:** 2026-10-16

**Authors:** Muhammad Khairul Anwar

**Affiliations:** 1 State University of Surabaya, Surabaya, Indonesia

This manuscript presents a relevant and well-structured bibliometric-systematic literature review examining the transition from compliance-based approaches toward collaborative financial governance in public financial management. The combination of bibliometric analysis and an in-depth thematic synthesis provides a useful foundation for understanding the development of this research area.

 Overall, I consider the manuscript to have academic merit and to make a useful conceptual contribution. However, a few minor clarifications would improve its methodological transparency and the precision of its interpretation:

1. The manuscript reports the inclusion of 265 articles for bibliometric analysis and 39 studies for in-depth thematic synthesis. Further clarification is needed regarding the criteria used to select these 39 studies from the broader dataset. Such clarification would strengthen the transparency and reproducibility of the review process.
2. The authors indicate that four reviewers participated in the screening process. A brief clarification of how screening decisions were coordinated and how disagreements, if any, were resolved would be useful.
3. The limitations section should provide a more comprehensive acknowledgment of the methodological boundaries of the review. The exclusive use of Scopus-indexed, English-language journal articles published between 2022 and 2026 may have limited the coverage of relevant literature. The absence of a reported formal methodological quality appraisal of the studies included in the thematic synthesis also warrants acknowledgment.
4. The proposed collaborative financial governance framework is a valuable contribution. However, the authors should ensure that statements describing a shift from compliance-based to collaborative governance are consistently presented as conceptual interpretations of the reviewed literature rather than as evidence of a directly observed institutional transformation.
5. The bibliometric findings are presented systematically and provide useful insights into the development of the research field. The interpretation of Figure 3 and Table 2 would benefit from a clearer distinction between author productivity and scholarly influence, as publication counts and citation indicators represent different aspects of academic contribution. The authors should also revise the caption of Figure 3 from "Most Relevant Author" to "Most Relevant Authors" to ensure grammatical accuracy and consistency.

**Overall Recommendation**

 The manuscript makes a valuable contribution to the literature on public financial management by integrating bibliometric evidence with a thematic synthesis of collaborative governance mechanisms. The proposed framework provides a useful conceptual foundation for future research. The points raised above primarily concern methodological clarification, interpretation, and presentation, and can be addressed without substantial revisions to the study. I therefore recommend Approved with Reservations.

Are the rationale for, and objectives of, the Systematic Review clearly stated?

Yes

Is the statistical analysis and its interpretation appropriate?

Not applicable

If this is a Living Systematic Review, is the ‘living’ method appropriate and is the search schedule clearly defined and justified? (‘Living Systematic Review’ or a variation of this term should be included in the title.)

Not applicable

Are sufficient details of the methods and analysis provided to allow replication by others?

Partly

Are the conclusions drawn adequately supported by the results presented in the review?

Yes

Reviewer Expertise:

Public Administration, Policy, and Governance

I confirm that I have read this submission and believe that I have an appropriate level of expertise to confirm that it is of an acceptable scientific standard, however I have significant reservations, as outlined above.

# Response

**Published:** 2026-10-26

**Authors:** Simon Saimima

**Affiliations:** Public Administration, Universitas Brawijaya, Malang, East Java, Indonesia

We thank the reviewer for the constructive comments and suggestions. We have revised the manuscript accordingly, as detailed below.

**Comment 1:** Clarify the criteria used to select 39 studies from the 265 articles included in the bibliometric analysis.

**Response: **We have revised Section 3.6 to explain the selection criteria for the 39 studies, including their relevance to public financial governance and the six governance domains.

**Comment 2:** Explain how the four reviewers coordinated screening decisions and resolved disagreements.

**Response: **We have clarified the screening procedure in Section 3.6, including the use of common eligibility criteria and consensus discussions to resolve differences in judgment.

**Comment 3: **Expand the limitations concerning database coverage, language, publication period, and methodological quality appraisal.

**Response: **We have expanded the limitations in Section 7 to acknowledge the restrictions of using Scopus, English-language articles, and the 2022–2026 period. We also acknowledge that no formal methodological quality appraisal was conducted.

**Comment 4:** Clarify that the proposed shift toward collaborative financial governance is a conceptual interpretation.

**Response:** We have revised the relevant statements throughout the manuscript to clarify that the proposed framework reflects our interpretation of the reviewed literature and does not establish an observed institutional transformation.

**Comment 5:** Distinguish author productivity from scholarly influence and correct the caption of Figure 3.

**Response: **We have revised Section 4.2 to distinguish publication counts from citation indicators. The interpretations of Figure 3 and Table 2 have been clarified, and the Figure 3 caption has been corrected to "Most Relevant Authors."
